# Biodegradable Stents—A New Option for Benign Central Airway Stenosis

**DOI:** 10.1111/resp.70117

**Published:** 2025-08-28

**Authors:** Oğuz Karcıoğlu, Eda Burcu Boerner, Faustina Funke, Jane Winantea, Filiz Oezkan, Rudiger Karpf‐Wissel, Kaid Darwiche

**Affiliations:** ^1^ Faculty of Medicine, Department of Chest Diseases Hacettepe University Ankara Turkey; ^2^ Department of Interventional Pneumology Ruhrlandklinik—University Medicine Essen, University Duisburg‐Essen Essen Germany

**Keywords:** airway narrowing, bronchial stent, bronchoscopy, interventional pulmonology, stricture, tracheal stenosis

## Abstract

**Background and Objective:**

Patients diagnosed with benign central airway stenosis who are ineligible for surgical intervention require airway stents. The high complication rates associated with conventional silicone and metallic stents have led to the development of new devices with lower complication rates and easier insertion and removal. This paper presents our results, including the indications, patient characteristics, and outcomes.

**Methods:**

We reviewed patients who underwent bronchoscopy for airway stenosis due to a benign cause between January 2015 and February 2023 in the interventional pulmonology unit of a tertiary university hospital. The causes and locations of stenosis, outcomes, and complications were analysed in patients who received a minimum of one biodegradable (BD) stent. All procedures were performed under general anaesthesia using a rigid bronchoscope.

**Results:**

A total of 136 BD stents were implanted at 22 airway sites in 18 patients, with a median age of 56. Thirteen patients, three with prior metal stents and 10 with prior silicone stents, had a history of non‐BD stent usage. Twelve procedures (54.5%) used bronchial stents, whereas 10 procedures (45.4%) used tracheal stents. The median duration of BD stent use was 10.6 months (range: 0.1–72.0 months). Early complications included one moderate granulation formation and two dislocations that necessitated stent fixation: one using clips and the other sutured to an additional stent.

**Conclusion:**

The study indicates that BD stents are both safe and feasible for treating benign stenosis, offering a safer alternative to silicone and metallic stents while providing personalised treatment for patients.

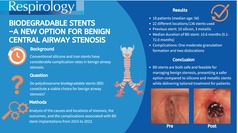

## Graphical abstract

Airway stenting using conventional stents for benign central airway stenosis has considerable complication rates as well as technical difficulties while placing and removing. Polydioxanone biodegradable stents serve as a feasible alternative for individuals requiring temporary airway support.

## 1 Introduction

Central airway stenosis (CAS) in adult patients, which can be caused by both malignant tumours and benign etiologies, typically leads to dyspnea, difficulty clearing secretions, coughing, and recurrent infections. The most common benign etiologies include idiopathic subglottic stenosis, prolonged intubation or tracheostomy, tracheomalacia, relapsing polychondritis, granulomatosis polyangiitis, and scarring after bronchoplastic surgery or transplantation [1, 2, 3, 4, 5]. Although non‐interventional therapies such as breathing exercises, airway clearance procedures, and pulmonary rehabilitation may improve symptoms in some patients, the majority often require repeated bronchoscopic interventions [6].

Although surgery is a definitive treatment modality for individuals with benign airway stenosis, its use is limited to subglottic stenosis, and its prognosis may not meet the initial expectations. Surgery entails specific perioperative and postoperative risks, including infection, bleeding, pneumomediastinum, subcutaneous emphysema, restenosis, and even death [7, 8, 9, 10]. It is essential to perform a comprehensive preoperative evaluation of underlying comorbidities, particularly cardiopulmonary comorbidities. The type of stenosis (presence of intraluminal lesions or scar tissue, tracheomalacia, or extrinsic compression), its location (subglottic, tracheal, or bronchial), length, and severity of the stenosis are also critical in determining whether the patient is an appropriate candidate for surgery or if an interventional approach is preferred. Significant surgical experience in the treatment of benign airway stenosis is necessary but is not widely available.

Several techniques are available for interventional bronchoscopy, including dilatation with a rigid bronchoscope or balloon, laser therapy, cryotherapy, argon plasma coagulation, electrocautery, and airway stenting [10]. This can help treat both short‐ and long‐term cases of airway stenosis. In cases of benign CAS, silicone stents are most commonly used because they are safe, well‐tolerated, and easy to place and remove [11]. However, their significant migration rate, mucus retention, and granulation tissue formation limit their use and acceptance by patients [10, 12]. Metallic stents are not recommended for benign airway stenosis because they are difficult to remove and cause restenosis due to the growth of granulation tissue (uncovered stents) and airway perforation [13, 14].

Biodegradable (BD) stents are self‐expanding devices made of an absorbable polymer (polydioxanone) that can be individually customised. They provide a low radial force that disappears with stent degradation within 3–4 months. BD stents are currently used to treat oesophageal and biliary stenosis and strictures [15, 16]. However, limited experience exists regarding their application in benign CAS [5]. In this study, we aimed to present our experience with BD stents for the treatment of benign airway stenosis.

## 2 Methods

### 2.1 Study Design

We retrospectively reviewed the patients who underwent bronchoscopy due to airway stenosis between January 1, 2015, and February 28, 2023, in the interventional pulmonology unit of the Ruhrlandklinik, a tertiary university hospital. We searched the terms ‘tracheal adhesion’, ‘tracheal stenosis’, ‘acquired stenosis of the trachea’, ‘acquired tracheal deformity’, ‘tracheomalacia’, ‘tracheal compression’, ‘tracheal collapse’, ‘tracheal cyst’, ‘tracheal abscess’, ‘bronchomalacia’, ‘bronchial stenosis’, and ‘other acquired tracheal disease’ to capture all relevant patients. Patients who underwent at least one stent placement were reviewed. We excluded patients who underwent only mechanical interventions, including argon‐plasma coagulation, electrocautery, cryotherapy, and balloon dilatation. Subsequently, we searched for patients with BD stents. Demographic and clinical data, pathological findings, other stents that may have been used before or after the BD stent, and the total duration of BD stent use were collected from the hospital database.

**FIGURE 1:**
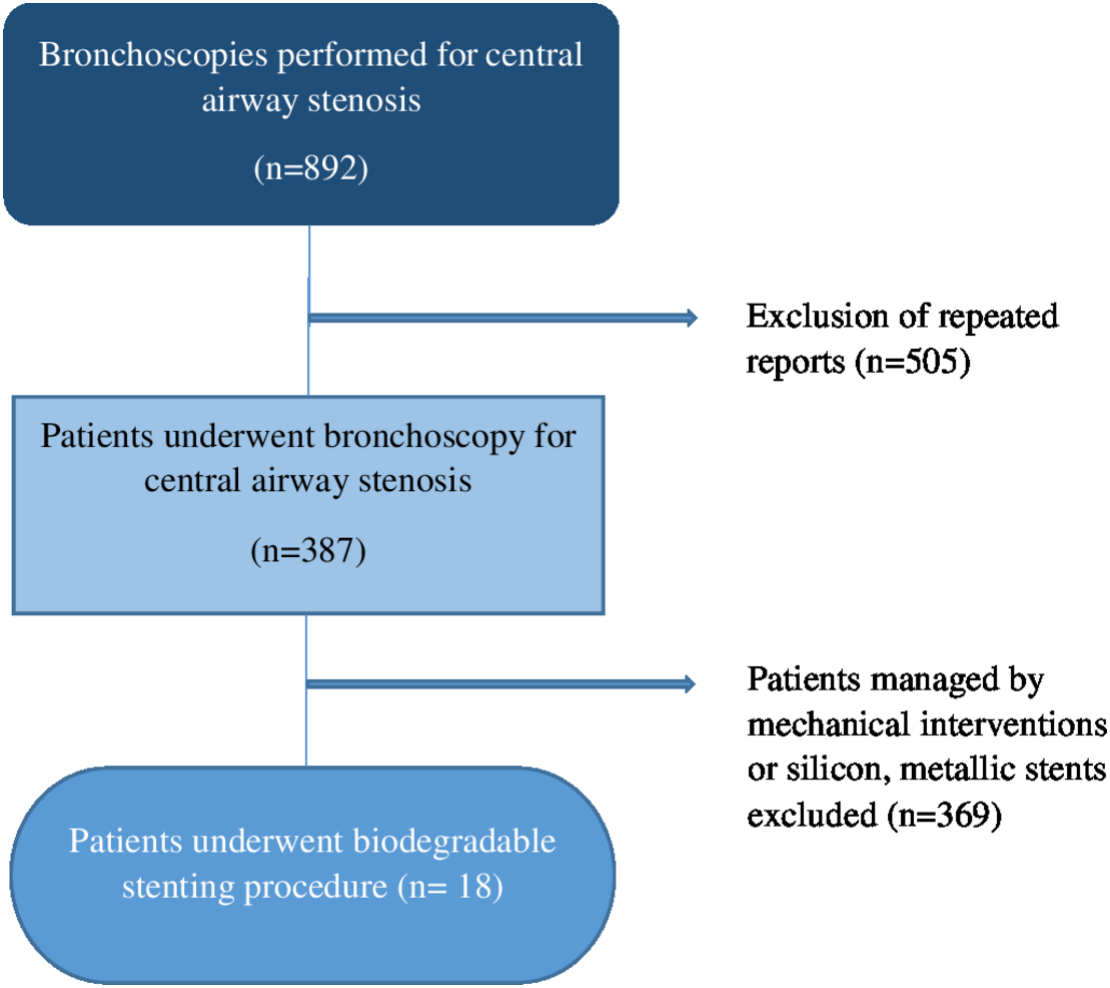
Study design.

The first day of BD stent use was defined as the first day of the BD stenting procedure, and the last day was 3 months after implantation if the stent was not removed or replaced with another BD or non‐BD stent, or if the patient had not died. If a non‐BD stent was used temporarily during BD stent use, and a BD stent was subsequently placed again, the durations were calculated and added separately. When there was a gap of more than 3 months between the two BD stenting procedures, each stent was considered to have a lifespan of 3 months unless restenting was postponed owing to bronchoscopy findings indicating that the preceding stent was stable. We defined the early period as the first month after the procedure, the subacute as 1–3 months, and the chronic period as beyond 3 months (Figure 1).

We assessed the intensity of granulation tissue development using three primary categories: mild, moderate, and severe. We characterised mild changes as slight enhancement of the mucosa, causing no symptoms and requiring no intervention. Moderate changes were defined as mucosal enhancement resulting in obstruction, necessitating interventions such as cryotherapy. Severe changes were described as causing scarring, subsequently leading to stenosis and necessitating interventions that included knife and balloon dilatation.

### 2.2 Bronchoscopic Procedures

Bronchoscopies were performed by experienced bronchoscopists (> 300 interventions). All procedures were performed under general anaesthesia using a rigid bronchoscope (Storz, Germany). All stents were placed using a dedicated delivery catheter, and the stent position was controlled using a standard videobronchoscope (Olympus Medical Systems, Japan). Fluoroscopy was not performed in any case. If the position of the stent is inappropriate, adjustment is easily possible using flexible or rigid forceps.

### 2.3 Statistical Analysis

We performed a univariate analysis for descriptive statistics and provided continuous variables as either mean (standard deviation) or median (minimum‐maximum), independent of the normality assumption. Data were presented as frequencies (%) of categorical variables. The normality assumption was assessed using the Shapiro–Wilk test, histograms, and boxplots. IBM SPSS Statistics for Windows, Version 23.0 (IBM Corp., Armonk, NY) was used for the statistical analysis.

## 3 Results

A total of 892 bronchoscopies were performed for central airway stenosis in 387 patients. In total, 136 BD stenting procedures were performed at 22 airway locations in 18 patients. Ten patients were male and eight were female. The median age was 56.0 (min: 21.5, max: 84.8) at the time of first BD stenting. The most common comorbidities were hypertension (seven patients), diabetes mellitus (five patients), chronic obstructive pulmonary disease (COPD), osteoporosis, and a history of a malignant tumour (three patients each), as shown in Table 1.

**TABLE 1:** Demographics and clinical information.

Number of patients	18	
Number of localizations	22	
Age (min–max)	56.0 (21.5–84.8)	
Female (*n*, %)	8 (44.4)	
Comorbidities (*n*, %)	Hypertension	9 (52.9)
	Diabetes mellitus	5 (29.4)
	COPD	3 (17.7)
	Osteoporosis	3 (17.7)
	OSAS	3 (17.7)
	ILD	2 (11.8)
	Pulmonary hypertension	2 (11.8)
	CAD	2 (11.8)
	Hypotyroidisim	2 (11.8)
	Reflux	2 (11.8)
	Granulomatous polyangiitis	2 (11.8)
	α‐1 antitripsin deficiency	1 (5.9)
	Asthma	1 (5.9)
	CVD	1 (5.9)
	Multiple Sclerosis	1 (5.9)
	CKD	1 (5.9)
	Dislipidemia	1 (5.9)
Cancer (n)	Lung (1)	
	Oropharyngeal (1)	
	Nasal (1)	
Transplantation	Lung (1)	
	Liver (1)	

Abbreviations: CAD: coronary artery disease; CKD: chronic kidney disease; COPD: chronic obstructive pulmonary disease; CVD: cerebrovascular disease; ILD: interstitial lung disease; max: maximum; min: minimum; n: number; OSA: obstructive sleep apnea.

The most common reason for stenting was tracheomalacia in five patients (27.7%) and polychondritis in four patients (22.2%). In 13 procedures, there was a previous history of non‐BD stent use, 10 of which were silicone and three of which were metal.

Tracheal and bronchial stents were used in 10 (45.4%) and 12 (54.5%) procedures, respectively. Of the latter, 9 (40.1%) were placed in the left main bronchus, 2 (9.1%) in the right intermediate bronchus, and 1 (4.5%) in the left upper lobe bronchus. The median duration of BD stent use was 10.6 months (min: 0.1‐max: 72.0), and the median number of stents for each patient was 3.5 (min: 1.0, max: 36.0), shown in Table 2.

**TABLE 2:** Details about stents.

Pre‐used stent (*n*, %)	13 (59.1%) Dumon silicone stent (10) Leufen stent (2) Advanta stent (1)
Indication for stenting (*n*, %)	Tracheomalacia 5 (27.7%)
	Polycondiritis 4 (22.2%)
	Tracheostomy 2 (11.1%)
	Prolonged intubation 2 (11.1%)
	Granulomatous Polyangiitis 2 (11.1%)
	Post‐transplantation narrowing 1 (5.5%)
	Scarring due to previous stenting 1 (5.5%)
	Foreign body 1 (5.5%)
Type of stent (localizations) (*n*, %)	Tracheal 10/22 (45.4%)
	Bronchial 12/22 (54.5%)
Localization	Trachea 10/22, (45.4%)
	Left main bronchus 9/22, (40.1%)
	Right intermediate bronchus 2/22, (9.1%)
	Left upper lobe bronchus 1/22, (4.5%)
Total duration with BD stent (months)	10.6 (min‐max: 0.1–72.0)
Total number of BD stents	3.5 (min‐max: 1.0–36.0)

Abbreviations: BD: biodegradable; n: number.

**FIGURE 2:**
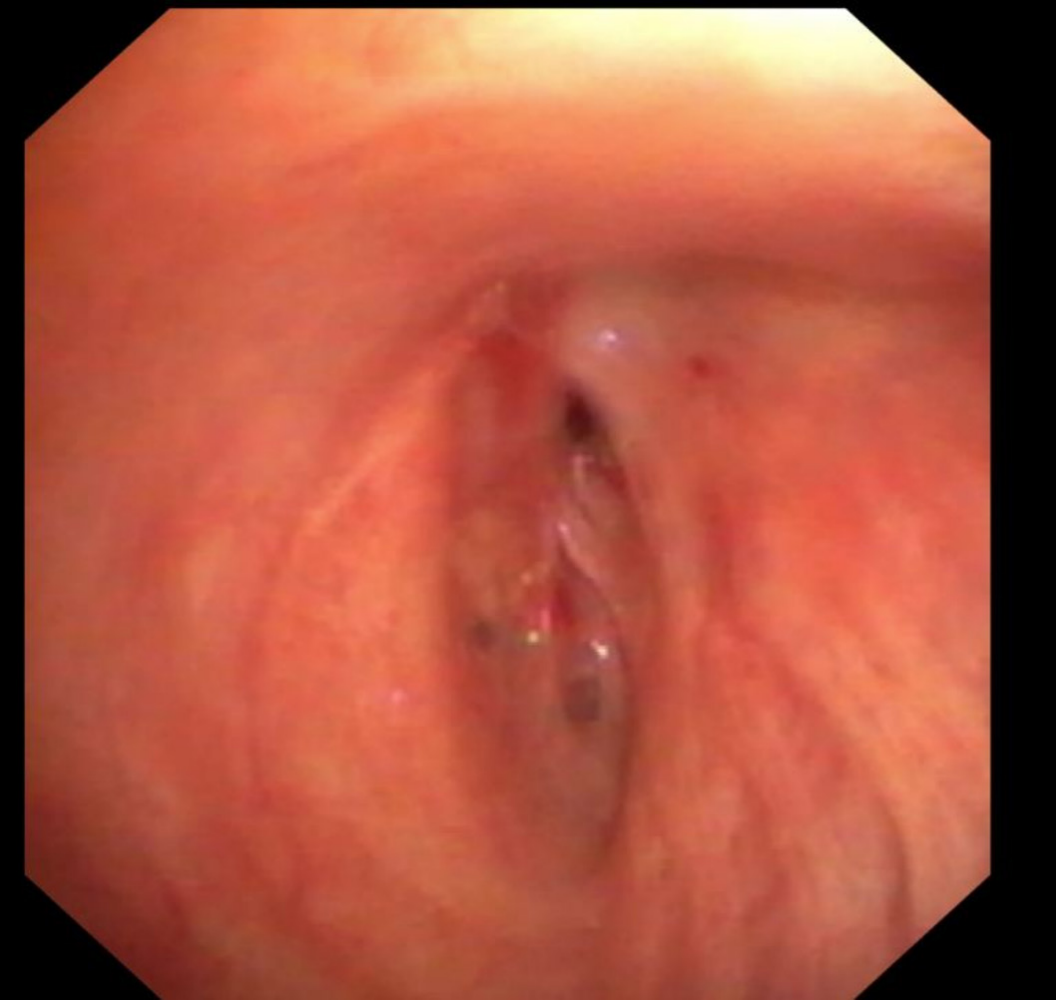
Bronchus intermedius stenosis in a patient after transplantation.

**FIGURE 3:**
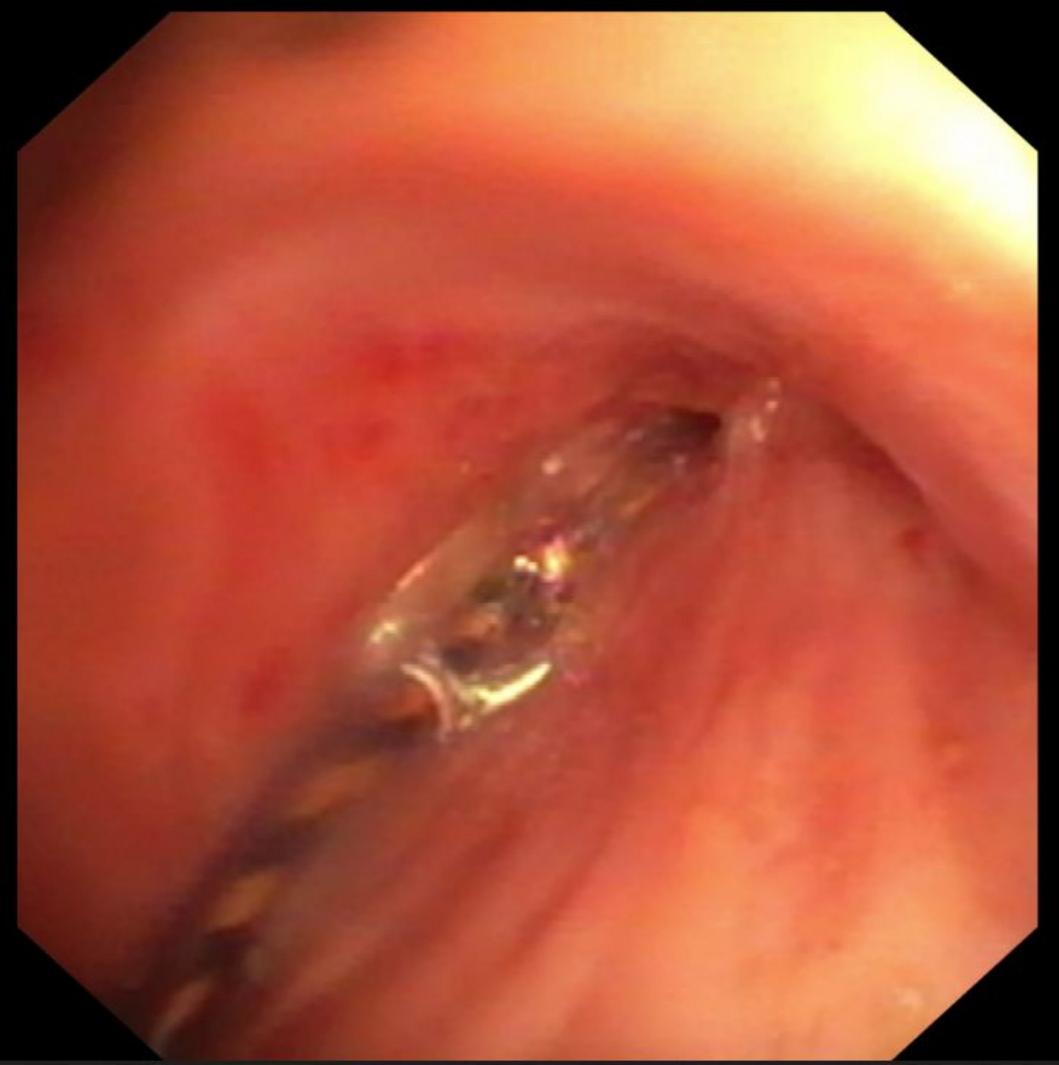
Careful insertion of the balloon into the stenosis in the bronchus intermedius.

**FIGURE 4:**
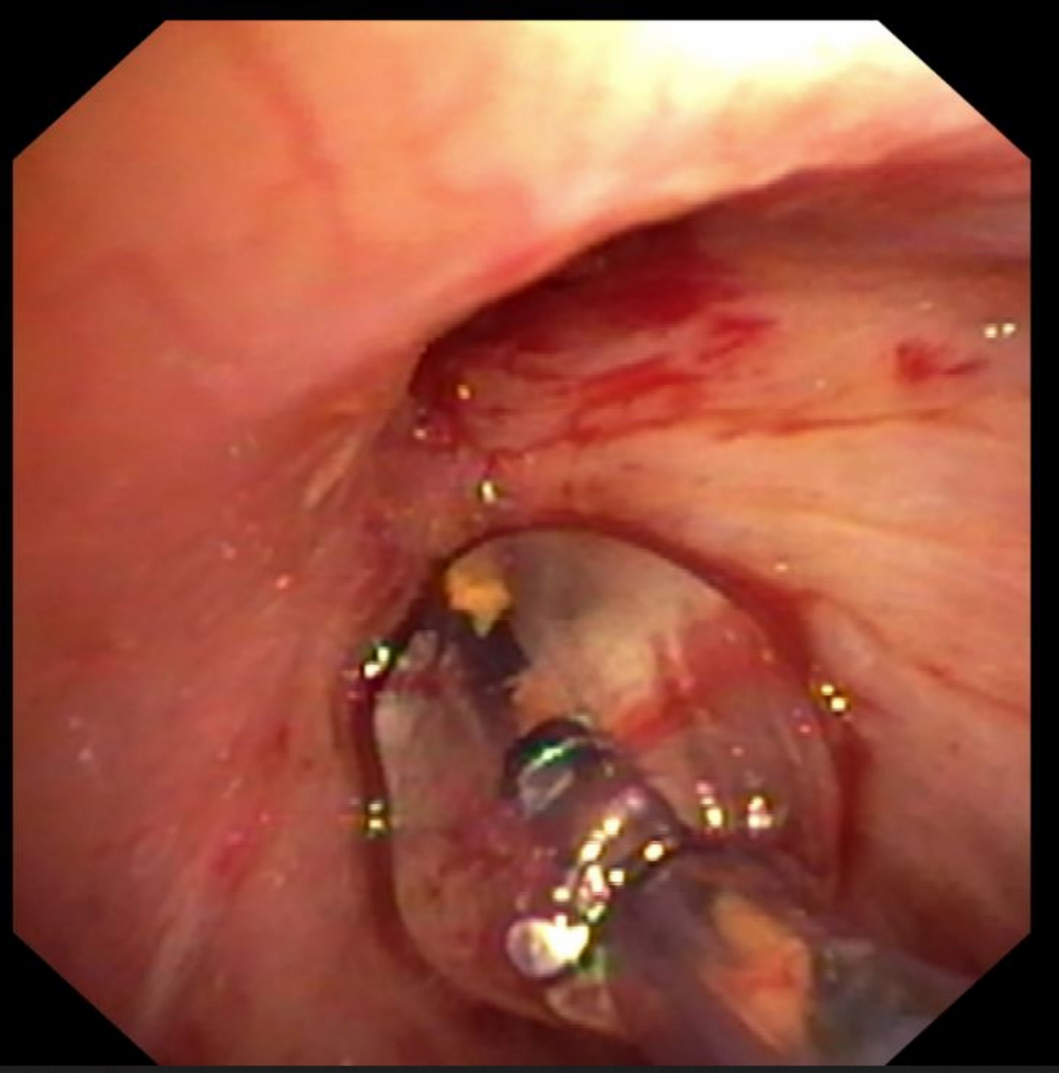
Balloon dilatation of bronchus intermedius.

**FIGURE 5:**
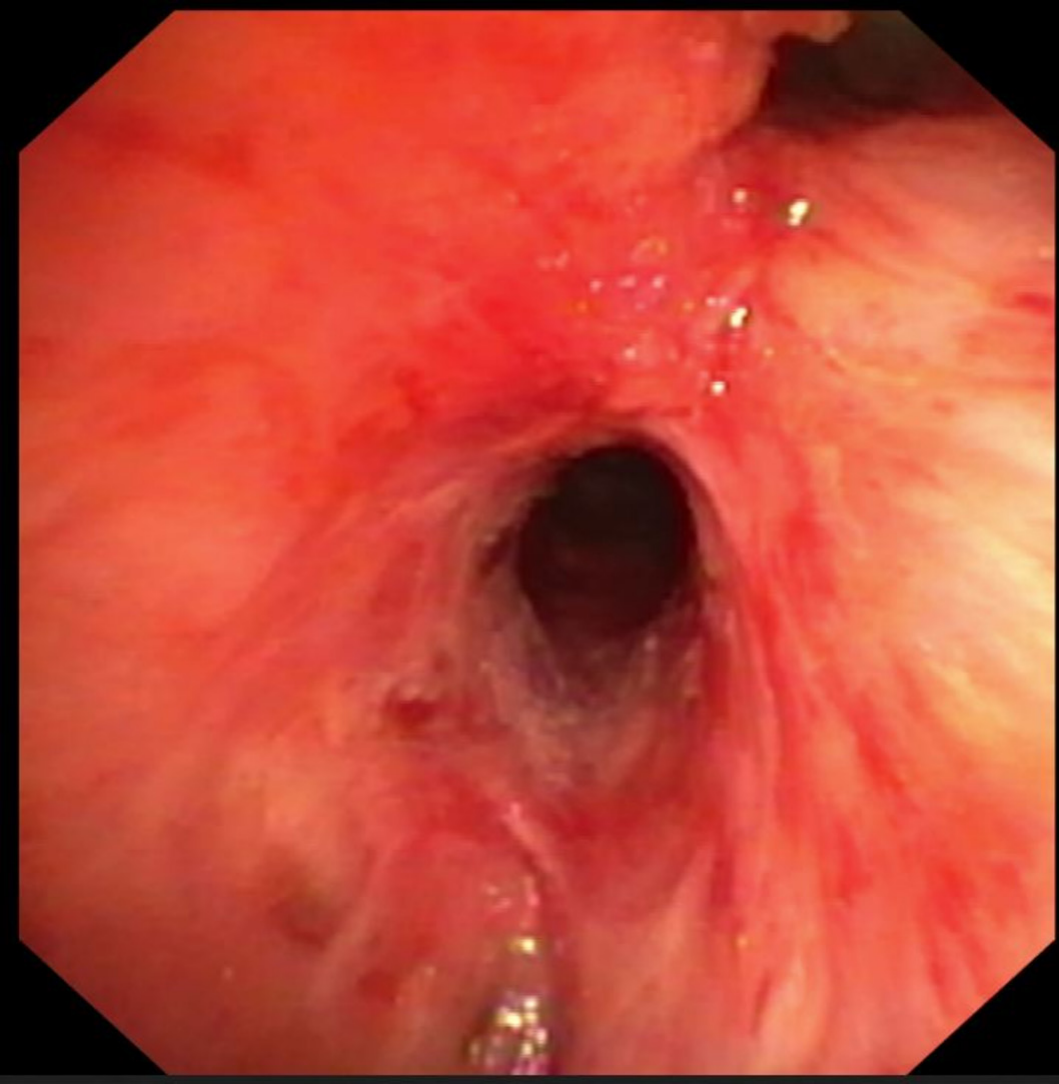
Bronchus intermedius after dilatation.

**FIGURE 6:**
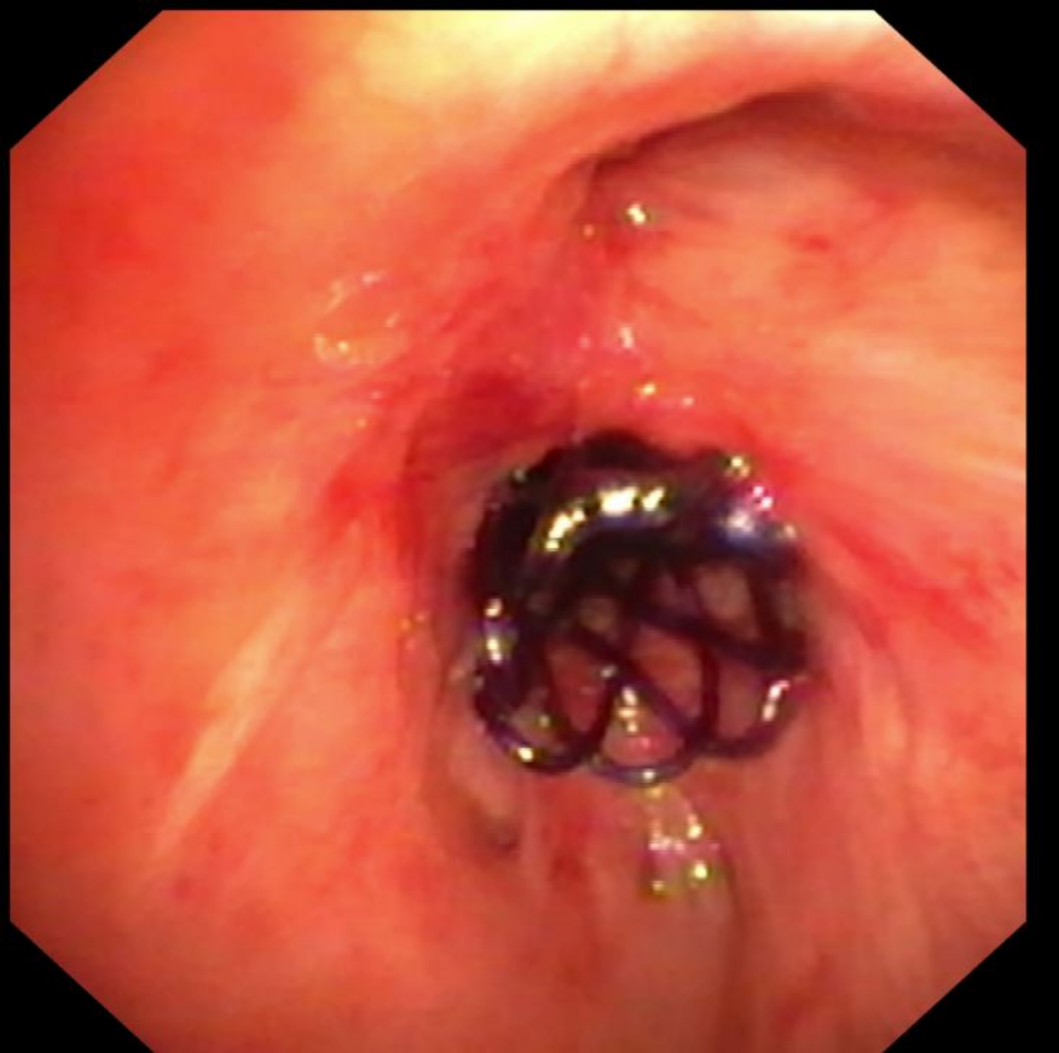
Biodegradable stent after implantation in bronchus intermedius.

Stent placement was technically successful in all cases without immediate complications. Early complications were observed in four interventions (4%) affecting three different patients. Two dislocations required stent fixation (one with clips and one sutured to another stent), one case of secretion retention required additional bronchoscopy, and one case of moderate granulation formation required recanalisation with cryotherapy. No further complications were recorded, including death, need for ventilation or ICU care, pre‐termed stent removal, or the need to change to a non‐BD stent. Four patients (23.5%) experienced granulation formation during the subacute period, and one patient underwent stent removal. Granulation formation occurred in five patients (29.4%) across seven locations (33.3%) as a chronic complication, as shown in Table 3. In patients who developed mild granulation formation in the subacute period, the mild granulation formation was noticed in the chronic period, too (Figures 2, 3, 4, 5, 6).

**TABLE 3:** Complications.

Acute^a^	Intervention	Subacute^a^	Intervention	Chronic^a^	Intervention
Dislocation (2)	Stent fixation (one with clips, one with suture to another stent)	Granulation formation (4)	Mechanical intervention^b^ and recanalization (3) Removal of stent (1)	Granulation formation (7)	Mechanical intervention^b^ and recanalization
Excessive secretions (1)	Cleaning (1)				
Granulation formation (1)	Mechanical intervention^b^ and recanalization				

^a^ Some patients exhibited granulation formation across more than one time intervals.

^b^ Mechanical interventions: Electric knife, argon plasma coagulation, cryoextraction, and balloon dilatation.

As a complication, eight cases (69.2%) exhibited mild granulation tissue, whereas four cases (30.8%) demonstrated moderate granulation requiring cryotherapy; no severe cases were observed (Table 4). Two patients with granulomatous polyangiitis and one patient post‐transplant exhibited significant scarring and severe airway narrowing, irrespective of the stent type employed. In these three patients, granulation tissue development was mild.

**TABLE 4:** Details on granulation formation.

Time of granulation tissue formation^a^	Degree	Intervention	Underlying disease	Previous non‐BD stents
Acute	Moderate	Cryotherapy	Tracheobronchomalacia	No
Subacute	Mild	None	Polychondritis	Yes
Subacute	Mild	None	Granulomatous Polyangiitis	Yes
Subacute	Moderate	Cryotherapy	Polychondritis	No
Subacute	Mild	None	Polychondritis	Yes
Chronic	Moderate	Cryotherapy	Polychondritis	No
Chronic	Mild	None	Post‐transplantation narrowing	Yes
Chronic	Mild	None	Prolonged intubation	Yes
Chronic	Mild	None	Polychondritis	Yes
Chronic	Moderate	Cryotherapy	Polychondritis	Yes
Chronic	Mild	None	Polychondritis	Yes
Chronic	Mild	None	Granulomatous Polyangiitis	Yes

Abbreviation: BD: biodegradable.

^a^ Some patients exhibited granulation formation across more than one time interval.

## 4 Discussion

The findings of this study indicate that BD stents may be an option for patients with benign airway stenosis who do not benefit from other stents or interventions such as dilatation and electrocautery and are not suitable candidates for surgery. Tracheomalacia is the most common cause of benign airway stenosis. The development of granulation tissue has emerged as a primary problem in acute, subacute, and chronic processes. We used tracheal stents in approximately half of the cases and bronchial stents in the majority of the remaining cases to manage the left main bronchial stenosis.

BD stents, a relatively novel tool for airway stenosis, provide an alternative for patients with benign airway stenosis who have a history of treatment failure with silicone and metallic stents [17]. Plojoux et al. reported that silicone and metallic stents were successfully used in 51% of the patients with dynamic A‐shaped tracheal stenosis due to intubation or tracheostomy [18]. Migration is one of the main adverse outcomes of previous stent placements. Numerous studies of both silicone and metallic stents have reported high migration rates [19, 20, 21]. Stehlik et al. argued that BD stents may be efficacious in managing benign airway stenosis due to their uncovered architecture, which aligns with the airway anatomy and minimises migration [22]. They experienced four dislocations among 62 stent implantations, with two cases requiring repositioning and fixation. The main disadvantage of BD stents has been reported to be their susceptibility to deformation when rigid instruments are used during dislocation management. Our experience with a few dislocations supports the idea that BD stents are a viable treatment option for benign airway stenosis.

Granulation is a major problem associated with this type of stent. The traditional approach to airway stenting is to use stents that are either fully compatible with the airway diameter or 10% larger to prevent migration, which is one of the main complications of airway stenting, especially for silicone stents [23]. In contrast, the use of larger stents produces more pressure on the mucosa, leading to increased granulation tissue formation [23, 24]. Theoretically, the low radial force of BD stents is expected to result in fewer inflammatory reactions and less granulation development due to the decreased pressure on the airway mucosa. In contrast, Stehlik et al. reported granulation formation in 23% of patients in the first 2 months and 47% thereafter. They suggested that this may be related to the use of the same diameter stent for the trachea with varying degrees of narrowing [22]. Our investigation revealed granulation formation in one procedure during the acute phase, four during the subacute phase, and seven during the chronic phase. Careful measurement of the diameter of the narrowed airway and use of customised stents may reduce granulation formation without causing migration.

Given that non‐BD stents often induce granulation formation, it can be assumed that such formation in patients may be associated with prior stent use. However, in our investigation, granulation was observed in patients with and without prior stenting, with no significant difference between the two groups. Significant airway blockage may be life‐threatening in certain patients. Within our cohort, two patients with granulomatous polyangiitis and one patient with post‐transplantation experienced severe airway obstruction. However, granulation tissue formation was mild in these patients. Our experience indicates that scar tissue formation is attributable to the activity of the underlying disease rather than stent interactions. In our opinion, immunosuppressive therapy remains the primary treatment, in addition to airway dilatation.

BD stents have an approximately 3–4‐month lifespan until they completely dissolve [23, 25]. Therefore, BD stents are considered temporary solutions for individuals whose stenosis is expected to heal rapidly following an intervention or for those who need time to undergo surgery [17]. However, this duration may be prolonged by repeated placements if necessary. Previous studies have demonstrated that the recurrent placement of BD stents is both safe and efficacious [26, 27]. In our experience, the number of stents used per patient varied between one and 20. This shows that BD stents can be used in the long term with repeated implantations when necessary, particularly in cases where surgical intervention is not feasible and alternative stents are not well tolerated by patients.

While the trachea is affected in most cases with symptoms due to airway stenosis, stenoses in the main and lobar bronchi may also contribute to these symptoms. Although there are few reports, the successful use of BD stents in both tracheal and bronchial stenoses shows promise for the management of benign airways [22, 25, 28]. In our study, stenting was implemented in the main and distal bronchi in approximately 50% of patients. We believe that the customisation of BD stents for stenosis is a primary advantage.

In experienced centres, BD stent implantation is almost indistinguishable from other stents. Rigid bronchoscopy under general anaesthesia is required for the use of silicone and metallic stents. Although it is theoretically possible to apply certain metallic stents with a fibreoptic bronchoscope, this is difficult to identify as a disadvantage of BD stents because most bronchoscopists prefer to use rigid bronchoscopy to ensure airway patency [29].

A great advantage of BD stents is that they are not only easy to place without serious complications but also have an uncovered design, which results in less retained secretions and better acceptance by patients in clinical practice. Additionally, the soft structure may be a significant advantage, allowing it to adapt entirely to the bronchial architecture. Their low radial force limits the pressure on the mucosa, which is beneficial for granulation formation. However, the reticulated structure attaches to the bronchial mucosa and facilitates external fixation [22].

Our study had some limitations. First, this was a retrospective study that analysed the clinical outcomes of individuals with a history of different previous stents or other interventions, with a diverse variety of causes of benign airway stenosis. As a significant proportion of patients were referred from different medical centres, it is possible that there were gaps in past treatments. In addition, because it is a life‐threatening condition requiring immediate treatment as soon as possible, it was not possible to define a control group. Conversely, the inclusion of detailed, real‐world data spanning approximately 60 months in a considerable number of patients strengthened our study.

In this study, we found that the use of BD stents was safe and feasible for treating benign stenosis. The safety profile of BD stents appears comparable to, or more favourable than, that of silicone and metallic stents.

In conclusion, uncovered BD stents are a safe and effective alternative for patients with benign airway stenosis, without life‐threatening complications. They offer individualised treatment for patients who are unable to tolerate conventional stents.

## Author Contributions

**Oğuz Karcıoğlu:** conceptualization (equal), data curation (equal), investigation (equal), methodology (equal), writing – original draft (lead). **Eda Burcu Boerner:** investigation (equal), methodology (equal), supervision (equal), writing – review and editing (equal). **Faustina Funke:** data curation (equal), resources (equal), software (equal). **Jane Winantea:** supervision (equal), visualization (equal). **FFiliz Oezkan:** supervision (equal). **Rudiger Karpf‐Wissel:** formal analysis (equal), investigation (equal), validation (equal). **Kaid Darwiche:** conceptualization (equal), supervision (equal), writing – original draft (equal).

## Ethics Statement

The study protocol adhered to the principles of the Declaration of Helsinki. The Clinical Research Ethics Committee of University Hospital Duisburg‐Essen approved the study (24‐12277‐BO). Adult participant consent was not required because it is a retrospectively designed study conducted using the hospital's database.

## Conflicts of Interest

The authors declare no conflicts of interest.

## Data Availability

Authors may share the data with readers in case of a reasonable request.
